# Blows on the Head

**Published:** 1879-08

**Authors:** 


### BLOWS ON THE HEAD

May be treated with applications of either warm or cold water while waiting for the doctor. Accidents to the head are very common with children from falls, blows, etc. When serious the child should be put to bed and given a thorough dose of salts or castor-oil. The head should be bathed with cold or warm water, liquor or some stimulating liniment. The body should be kept warm, and mustard or other stimulating applications put to the feet, the object being to draw the blood away from the head and prevent inflammation. The child should keep the bed till this danger is passed. The permanent injury resulting to children from blows inflicted on the head as punishment, calls for the severest reprobation. Thousands of children are ruined in this way, and their lives made useless through suffering from diseases of the brain thus induced.

If a child is to be beaten (as it is not permitted to beat dumb brutes in any highly civilized community) they should never be struck on the head.

There are better methods of correcting children than to beat them, and I doubt if any child was ever made better by it. Some important element of humanity must be wanting in the parent or teacher who can seize the little frail form of a child that can make no resistance and inflict pain upon it with blows. I would as soon think of striking my father or mother as one of my children. My parents entertained the same views, though they had a houseful of noisy and happy youngsters to manage. Still none of us went to the bad. Indeed, I am willing to confess myself the worst of the seven.

